# Metabolic Flux Analysis Reveals the Roles of Stearate and Oleate on CPT1C-mediated Tumor Cell Senescence

**DOI:** 10.7150/ijbs.80822

**Published:** 2023-04-09

**Authors:** Panpan Chen, Jingyu Tian, Yanying Zhou, Yixin Chen, Huizhen Zhang, Tingying Jiao, Min Huang, Hui Zhang, Peng Huang, Ai-Ming Yu, Frank J. Gonzalez, Huichang Bi

**Affiliations:** 1 NMPA Key Laboratory for Research and Evaluation of Drug Metabolism, Guangdong Provincial Key Laboratory of New Drug Screening, School of Pharmaceutical Sciences, Southern Medical University, Guangzhou 510515, China.; 2 Guangdong Provincial Key Laboratory of New Drug Design and Evaluation, School of Pharmaceutical Sciences, Sun Yat-sen University, Guangzhou 510006, China.; 3 Department of Pharmacy, Shanghai Ninth People's Hospital, Shanghai Jiao Tong University School of Medicine, Shanghai 201999, China.; 4 Guangdong University of Technology, Guangzhou 510006, China.; 5 Sun Yat-Sen University Cancer Center, Guangzhou 510275, China.; 6 Department of Biochemistry and Molecular Medicine, Comprehensive Cancer Center, UC Davis School of Medicine, Sacramento, CA 95817, USA.; 7 Laboratory of Metabolism, Center for Cancer Research, National Cancer Institute, National Institutes of Health, Bethesda, MD 20892, USA.

**Keywords:** cellular senescence, carnitine palmitoyltransferase 1C (CPT1C), metabolic flux analysis, stearate, oleate

## Abstract

Cellular senescence is a state of proliferative arrest, and the development of carcinoma can be suppressed by conferring tumor cell senescence. Recently, we found that carnitine palmitoyltransferase 1C (CPT1C) controls tumor cell proliferation and senescence via regulating lipid metabolism and mitochondrial function. Here, ^13^C-metabolic flux analysis (^13^C-MFA) was performed and the results revealed that *CPT1C* knockdown in MDA-MB-231 cells significantly induced cellular senescence accompanied by altered fatty acid metabolism. Strikingly, stearate synthesis was decreased while oleate was increased. Furthermore, stearate significantly inhibited proliferation while oleate reversed the senescent phenotype induced by silencing *CPT1C* in MDA-MB-231 cells as well as PANC-1 cells. A939572, an inhibitor of stearoyl-Coenzyme A desaturase 1, had the same effect as stearate to inhibit cellular proliferation. These results demonstrated that stearate and oleate are involved in CPT1C-mediated tumor cellular senescence, and the regulation of stearate/oleate rate via inhibition of SCD-1 could be an additional strategy with depletion of CPT1C for cancer therapy.

## Introduction

Cellular senescence was originally found in normal human somatic cells that only divide a limited number of times and enter a state of cell proliferation arrest during culture 1, 2. Tumor cells or cancer cells can also enter into cellular senescence triggered by various factors such as therapeutic stimuli 3. Traditional cancer therapy often uses high doses of drugs or irradiation to rapidly kill dividing cancer cells via inducing extensive DNA damage which can cause serious side effects such as damage to neighboring normal cells, potentiating cancer recurrence and cancer cell resistance to therapy in part through the mutation of oncogenes and tumor suppressor genes 4. Recent work has provided substantial *in vivo* data supporting the view that cellular senescence could act through a tumor suppressor mechanism 5.

It is well established that energy metabolism reprogramming is an emerging hallmark of cancer that can fuel cell proliferation via the adjustment of energy metabolism 6. Elevated fatty acid synthesis has been considered a significant aberration of tumor cellular metabolism 7. Fatty acid is a carboxylic acid that mainly occurs in even carbon numbers and is either saturated or unsaturated 8. Fatty acids are important for energy storage, membrane genesis and homeostasis, and as substrates for the synthesis of signaling molecules 9. Targeting cancer metabolism, including fatty acid metabolism, is a new strategy for the treatment of malignancies 10.

The carnitine palmitoyltransferase (CPT) system, which consists of CPT1 and CPT2, controls long-chain acyl-CoA transport from cytoplasm to mitochondria 11. CPT1C, the last discovered subtype 12, plays an important role in energy homeostasis 13, 14. CPT1C can also promote tumor cell survival under metabolic stress 15, 16. Tumor tissue samples from patients with various cancers, especially breast cancer and pancreatic cancer, show higher CPT1C expression compared with normal tissue 15, 17-21. Targeting of CPT1C may offer an opportunity to modulate cancer cell metabolism and slow tumor growth 20. Most recently, we found that CPT1C is a crucial regulator of tumor cell proliferation and senescence and exerts the most prominent role compared with other CPT subtypes 19, 22, 23. CPT1C is also a novel target of peroxisome proliferator-activated receptor α (PPARα) and estrogen-related receptor α (ERRα), that control fatty acid metabolism 18, 24. Furthermore, CPT1C protects tumor cells from senescence and this activity is closely related to lipid metabolism 25. In normal MRC-5 cells, gain-of-function of CPT1C can also reverse cellular senescence via the regulation of lipid accumulation and mitochondrial function 26. Though it has been well demonstrated that *CPT1C* knockdown induces cellular senescence (Supporting Information Fig. S1) 22, 23, 25, the relationship between CPT1C and fatty acid (FA) metabolism during tumor cell senescence induced by silencing *CPT1C* is unclear.

In the current study, ^13^C-metabolic flux analysis (^13^C-MFA) was used to explore the relationship between CPT1C and fatty acid metabolism during tumor cell senescence. The ^13^C-MFA results indicated that *CPT1C* knockdown could significantly induce cellular senescence and alter fatty acid anabolism and catabolism in MDA-MB-231 cells. Moreover, stearate and oleate play a vital role in CPT1C-mediated tumor cell proliferation and senescence. Stearate can inhibit cellular proliferation while oleate can reverse the senescent phenotype induced by silencing *CPT1C*. Taken together, CPT1C and the regulation of stearate and oleate might be potential targets for cancer therapy.

## Materials and methods

### Cell culture

The human breast cancer MDA-MB-231 cell line was obtained from Dr. Jun Du's at Sun Yat-sen University and the human pancreas cancer PANC-1 cell line was purchased from Guangzhou Cellcook Biotech Company (Guangzhou, China). The cells were cultured in Dulbecco's modified Eagle's medium (DMEM, Corning, NY) containing 4.5 g/L glucose, L-glutamine and sodium pyruvate supplemented with 10% fetal bovine serum (FBS, Gibco, USA), 100 U/mL penicillin sodium and 100 μg/mL streptomycin sulfate (Gibco, USA) at 37 °C in a 5% CO_2_ water-saturated environment. Cell lines were authenticated by Guangzhou Cellcook Biotech by use of Short Tandem Repeat Authentication. The cells were treated with BSA conjugated-stearate, BSA conjugated-oleate or A939572 (SCD-1 inhibitor) at 24 h after siRNA transfection and incubated for another 48 h.

### Transfection with small interfering RNA

Small interfering RNA (siRNA) was used to decrease CPT1C level in MDA-MB-231 and PANC-1 cells. Cells were transfected with 50 nM siRNA or siControl (Ruibo Biotech, Guangzhou, China) using Lipofectamine RNAiMAX (Invitrogen, USA) with reduced serum medium Opti-MEM (Gibco, USA). Quantitative real-time PCR and western blot were used to measure the *CPT1C* mRNA and CPT1C protein levels, respectively, to validate the transfection efficiency. The specific human CPT1C siRNAs sequences are shown in Supporting Information Table S1.

### RNA extraction and quantitative real-time PCR analysis

RNA extraction and quantitative real-time PCR were performed as described in an earlier study 23. Total RNA was extracted by Trizol reagent (Invitrogen, USA). The RNA quantity and quality were evaluated by a NanoDrop Flex Station 3 spectrophotometer (Thermo, USA). RNA (one microgram) was purified and reverse-transcribed to cDNA by the PrimeScript RT reagent kit with gDNA eraser (TaKaRa, Japan). Real-time PCR was performed using TB Green Premix Ex Taq II kit (TaKaRa, Japan) in 7500 Real-Time PCR System (Applied Biosystems, USA) and the results were analyzed by the 2^-ΔΔCt^ method. The sequences of primers are shown in Supporting Information Table S2.

### Western blot analysis

Western blot analysis was performed according to a previous report 23. RIPA lysis buffer and the BCA protein assay kit (Thermo, USA) were used to extract and quantify cellular proteins. The proteins were then electrophoresed on SDS-PAGE and transferred to polyvinylidene fluoride membranes (Millipore, USA). After blocking, the membranes were incubated with different antibodies against CPT1C (Abcam, Cat# ab123794, RRID: AB _10974231), ME1 (Sangon Biotech, Cat# D261900, RRID: AB_2877135), ME2 (Sangon Biotech, Cat# D163864, RRID: 2877136), ME3 (Sangon Biotech, Cat# D153183, RRID: AB_2877137), CD36 (Sangon Biotech, Cat# D161529, RRID: AB_2877138), ACLY (Sangon Biotech, Cat# D221957, RRID: AB_2877139), FASN (Sangon Biotech, Cat# D262701, RRID: AB_2877140), SCD-1 (Sangon Biotech, Cat# D162163, RRID: AB_2877141) or GAPDH (Cell Signaling Technology, Cat# 2118, RRID: AB_561053) at 4 °C overnight. Then the immunoblot bands were visualized by use of anti-rabbit IgG horseradish peroxidase-linked secondary antibody (Cell Signaling Technology, Cat# 7074S, RRID: AB_2099233). The protein-antibody complexes were determined with an ECL detection kit (Millipore, USA) and the intensity of protein bands was measured by Quantity One software (Bio-Rad Laboratories, Hercules, USA).

### Senescence analysis

Cell proliferation ability was measured by ELISA assay (Roche, Switzerland). Cells were cultured with BrdU for 24 h, fixed *in situ* and incubated with the anti-BrdU antibodies for 90 min. Then cells were incubated with the substrate solution for another 5 min after rinsing with PBS. The absorbance was detected at 370 nm with reference wavelength at 492 nm. Colony formation ability was determined with Diff-Quik (Propbs, China). Cells were plated with low density (5000, 2500 or 1250) and cultured for about 2 weeks, and then cells were fixed and stained with Diff-Quik. SA-β-gal activity was detected at pH 6.0 using Senescence β-Galactosidase Staining Kit (Beyotime, China). Cells were fixed and then stained in staining working solution containing X-Gal overnight at 37 °C. The ratio of SA-β-gal positive cells was counted and photographs taken by use of a microscope (×200, Olympus, Japan).

### Tracer experiments

For [U-^13^C_16_] palmitate tracer experiments, 2.5 mM [U-^13^C_16_] sodium palmitate (Cambridge Isotopes, USA) was dissolved in 150 mM sodium chloride solution at 70 °C, and 40 mL of palmitate solution was added into 50 mL of 0.34 mM ultra-fatty acid free BSA (Sigma‐Aldrich, USA) solution at 37 °C to conjugate [U-^13^C_16_] palmitate to BSA. 1 mM working BSA-conjugated [U-^13^C_16_] palmitate solution was prepared via adjusting the pH to 7.4 and diluting to 100 mL with 150 mM sodium chloride. Finally, 50 µM BSA-conjugated [U-^13^C_16_] palmitate and 1 mM carnitine (Sigma‐Aldrich, USA) were mixed with culture medium in 10% dialyzed FBS. For [U-^13^C_6_] glucose tracer experiments, tracer media consisted of glucose free DMEM medium (Gibco, USA) with 10% FBS, supplemented with [U-^13^C_6_] glucose (Cambridge Isotopes, USA). During tracer experiments, the medium was removed, cultured cells were rinsed with PBS, and tracer media added to the wells. Cells were cultured in tracer media for 24 - 48 h before metabolite extraction.

### Metabolite extraction and derivatization

Polar metabolites and fatty acids were extracted using methanol/water/chloroform solution as described previously 27. Briefly, the medium was removed and cells washed with 0.9% (w/v) saline, and cellular metabolites quenched with 500 μL of ice-cold methanol. Then 200 μL of ice-cold water supplemented with 1 μg norvaline internal standard was added to each well. The solution and cells were transferred to new sample tubes, and 500 μL of ice-cold chloroform containing 1 μg hexadecanoic-D31 acid added. After vortexing and centrifugation, the polar metabolites (top aqueous layer) and lipids (bottom organic layer) were collected and dried under airflow. All reagents were obtained from Sigma-Aldrich.

For derivatization of polar metabolites, tp each tube 20 μL of 2% (w/v) methoxyamine hydrochloride (MP Biomedicals, USA) in pyridine was added and the samples incubated at 37 °C for 60 min. Subsequently polar metabolite tert-butyldimethylsilyl (tBDMS) derivatives were obtained by adding 30 μL of N-methyl-N-(tert-butyl-dimethylsilyl) trifluoroacetamide + 1% tert-butyldimethylchlorosilane (Regis Technologies, USA) and incubating at 37 °C for 30 min. For derivatization of fatty acids, dried fatty acids were dissolved in 500 μL of 2% (v/v) methanolic sulfuric acid (Sigma-Aldrich, USA) and held at 50 °C for 2 h. Then fatty acid methyl esters (FAMEs) were extracted in 500 μL of hexane with 100 μL of saturated sodium chloride.

### Gas chromatography/mass spectrometry analysis

Gas chromatography/mass spectrometry (GC/MS) analysis was performed using Thermo 1310 with a 30-m DB-35MS capillary column (Agilent Tech-nologies) connected to an ISQ QD MS. GC/MS was operated under the condition of electron impact (EI) ionization at 70 electronvolts (eV). The MS source was kept at 300 °C, and the detector was used under scanning mode with a recorded ion range of 100 - 650 mass-to-charge ratio (m/z). In splitless mode, 1 μL of sample was injected at 270 °C with helium as the carrier gas at the flow rate of 1.2 ml/min. For analysis of polar metabolites derivatives, the GC oven temperature was kept at 100℃ 2 min, and raised to 255℃ at 3.5 °C/min, then to 320 °C at 15 °C/min, with a total run time of about 50 min. For analysis of FAMEs, the GC oven temperature was kept at 100℃ 3 min, and raised to 205 °C at 25℃/min, further to 230 °C at 1.5 °C/min, then to 280 °C at 25 °C/min, with a total run time of about 29 min. To quantify the metabolites and mass isotopomer distributions, the selected ion fragments were integrated by Trace Finder 3.2 and corrected for natural isotope abundance. The metabolite fragments are listed in Table S3. Total abundance was normalized by the internal standard control.

### CCK8

Cell viability was analyzed by Cell Counting Kit-8 (Dojindo, Janpan). Briefly, cells were seeded into 96-well plates at a final volume of 100 μL per well. Treatment of BSA conjugated-stearate, BSA conjugated-oleate or SCD-1 inhibitor A939572 for 48 h, the cell activity was determined by addition of 10 μL of highly sensitive reagent to each well, incubation for 1 h at 37 °C and the absorbance was detected at 450 nm wavelength. The preparation method of BSA conjugated-stearate and BSA conjugated-oleate was the same as BSA-conjugated [U-^13^C_16_] palmitate.

### Statistical analysis

All results were presented as mean ± S.E.M.* P* values were calculated by the use of two-tailed student *t* test or One-way ANOVA followed by the Student - Newman - Keuls post hoc test. The graphs were prepared using GraphPad Prism 8.0 software (GraphPad Software Incorporated, USA).

## Results

### CPT1C knockdown alters anabolism and catabolism of exogenous fatty acids in MDA-MB-231 cells

**Figure 1:**
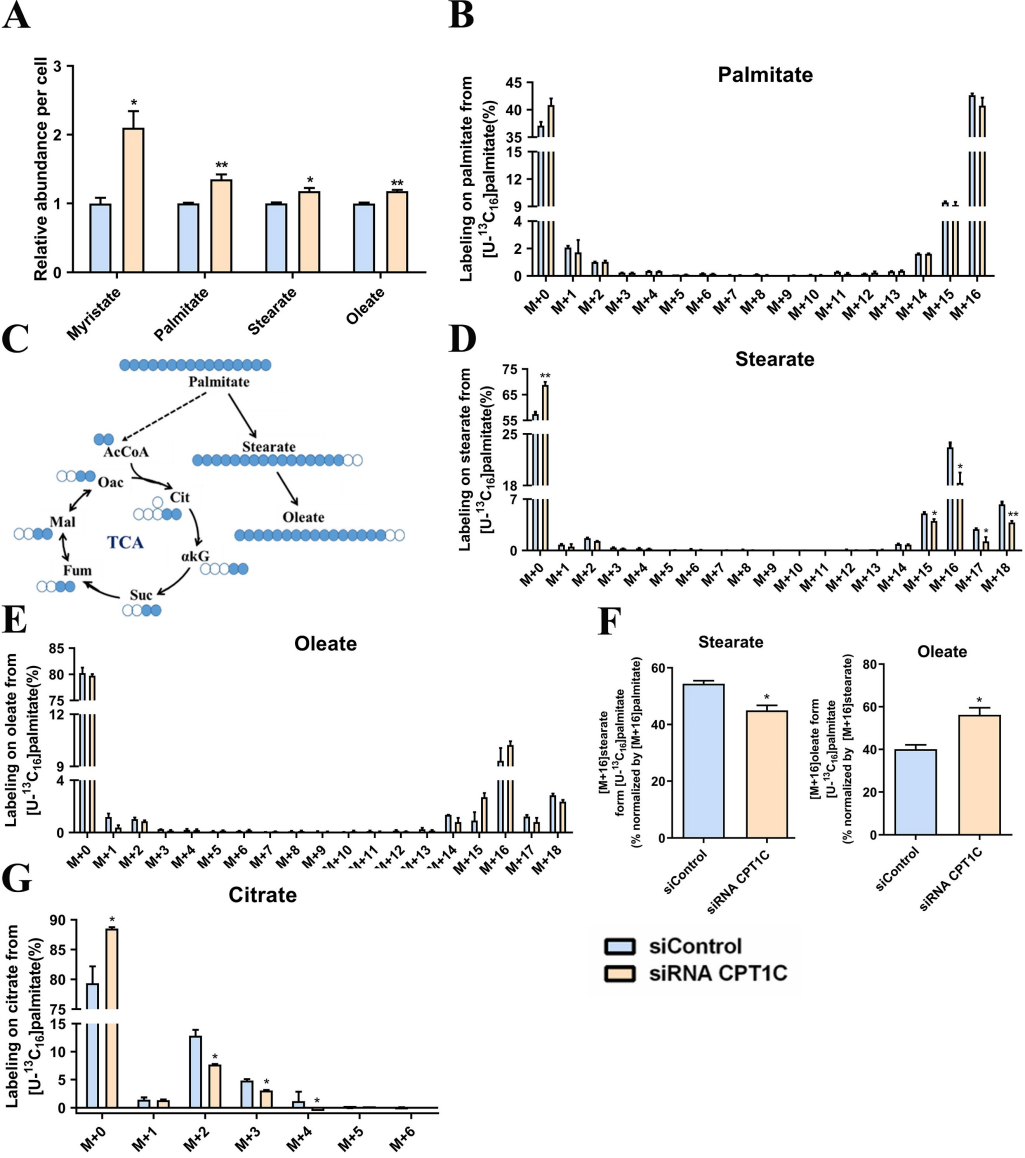
** CPT1C knockdown alters anabolism and catabolism of exogenous fatty acids in MDA-MB-231 cells. A** Relative fatty acids abundance in MDA-MB-231 cells was normalized by cell number, n=3. **B** Mass isotopomer distribution of palmitate in MDA-MB-231 cells cultured with BSA conjugated-[U-^13^C_16_] palmitate and 1 mM carnitine, n=3. **C** Schematic of [U-^13^C_16_] palmitate metabolism. Open circles depict ^12^C and filled circles depict ^13^C atoms. **D** Mass isotopomer distribution of stearate in MDA-MB-231 cells cultured with BSA conjugated-[U-^13^C_16_] palmitate and 1 mM carnitine, n=3. **E** Mass isotopomer distribution of oleate in MDA-MB-231 cells cultured with BSA conjugated-[U-^13^C_16_] palmitate and 1 mM carnitine, n=3. **F** Percentage of normalized newly synthesized fatty acids, left and right: stearate and oleate, n=3. **G** Mass isotopomer distribution of citrate in MDA-MB-231 cells cultured with BSA conjugated-[U-^13^C_16_] palmitate and 1 mM carnitine, n=3. Data are represented as mean ± S.E.M, ^*^p < 0.05, ^**^p < 0.01 versus the siControl group. AcCoA, acetyl-CoA; αKG, α-ketoglutarate; Cit, citrate; Fum, fumarate; Mal, malate; Oac, oxaloacetate; Suc, succinate.

To investigate the relationship between CPT1C and fatty acid metabolism, MDA-MB-231 cells were cultured in media supplement with BSA-conjugated [U-^13^C_16_] palmitate. Consistent with a previous report 25, *CPT1C* knockdown remarkably increased the total abundance of myristate, palmitate, stearate and oleate (Fig. 1A). In addition, MDA-MB-231 cells were highly dependent on exogenous BSA-conjugated [U-^13^C_16_] palmitate and *CPT1C* knockdown slightly decreased the abundance of [M+16] palmitate (Fig. 1B). Albumin-bound [U-^13^C_16_] palmitate is the sole source of fatty acid (Fig. 1C), and it is notable that [M+16] stearate was decreased markedly but [M+16] oleate was increased slightly after silencing *CPT1C* (Fig. 1D and 1E). Furthermore, *CPT1C* knockdown indeed decreased the synthesis rate of stearate and increased the synthesis rate of oleate notably (Fig. 1F). Besides, the abundance of labeled citrate from [U-^13^C_16_] palmitate was decreased markedly upon *CPT1C* knockdown (Fig. 1G). Overall, *CPT1C* knockdown could alter anabolism and catabolism of exogenous fatty acids in MDA-MB-231 cells.

### CPT1C knockdown alters anabolism and catabolism of endogenous fatty acids in MDA-MB-231 cells

**Figure 2:**
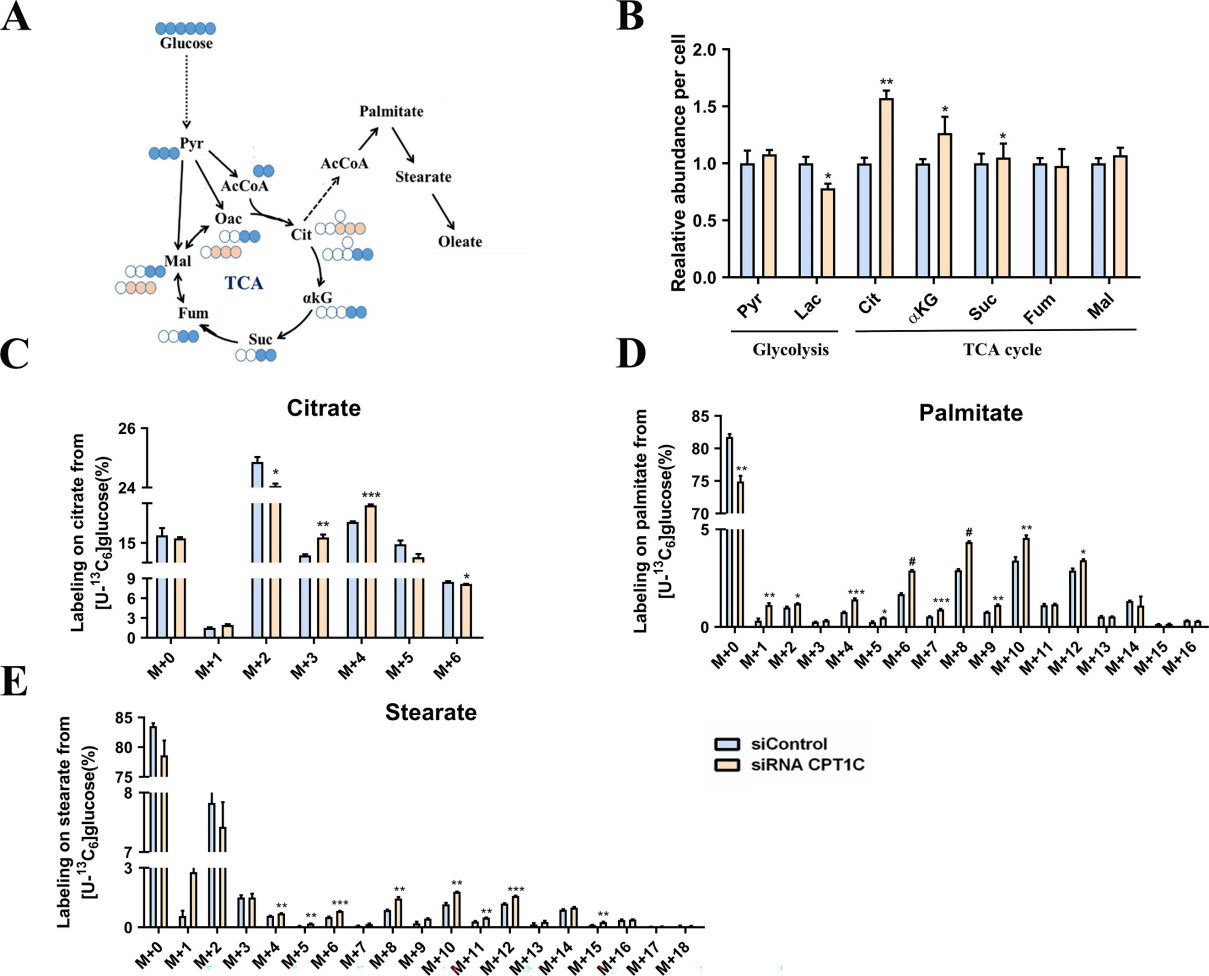
** CPT1C knockdown alters anabolism and catabolism of endogenous fatty acids in MDA-MB-231 cells. A** Schematic of [U-^13^C_6_] glucose metabolism. Open circles depict ^12^C and filled circles depict ^13^C atoms, filled pink circles indicate PC or ME pathway. **B** Relative metabolites abundance in MDA-MB-231 cells was normalized by cell number, n=3. **C** Mass isotopomer distribution of citrate in MDA-MB-231 cells cultured with [U-^13^C_6_] glucose, n=3. **D** Mass isotopomer distribution of palmitate in MDA-MB-231 cells cultured with [U-^13^C_6_] glucose, n=3. **E** Mass isotopomer distribution of stearate in MDA-MB-231 cells cultured with [U-^13^C_6_] glucose, n=3. Data are represented as mean ± S.E.M, ^*^p < 0.05, ^**^p < 0.01, ^***^p < 0.001, ^#^*p* < 0.0001 versus the siControl group. AcCoA, acetyl-CoA; αKG, α-ketoglutarate; Cit, citrate; Fum, fumarate; Mal, malate; Oac, oxaloacetate; Pyr, pyruvate; Suc, succinate.

[U-^13^C_6_] Glucose was next employed to quantify de novo lipogenesis in MDA-MB-231 cells (Fig. 2A). It's surprising that the total abundance of citrate was increased significantly after *CPT1C* knockdown (Fig. 2B). As shown in Fig. 2C, [M+2] and [M+6] citrates were decreased while [M+3] and [M+4] citrates were markedly increased (Fig. 2C). Moreover, the abundance of labeled palmitate and stearate from [U-^13^C_6_] glucose were significantly increased upon *CPT1C* knockdown (Fig. 2D and 2E). In short, *CPT1C* knockdown could alter anabolism and catabolism of endogenous fatty acids in MDA-MB-231 cells.

### CPT1C knockdown affects expression of genes and proteins related to citrate and fatty acid metabolism in MDA-MB-231 cells

**Figure 3:**
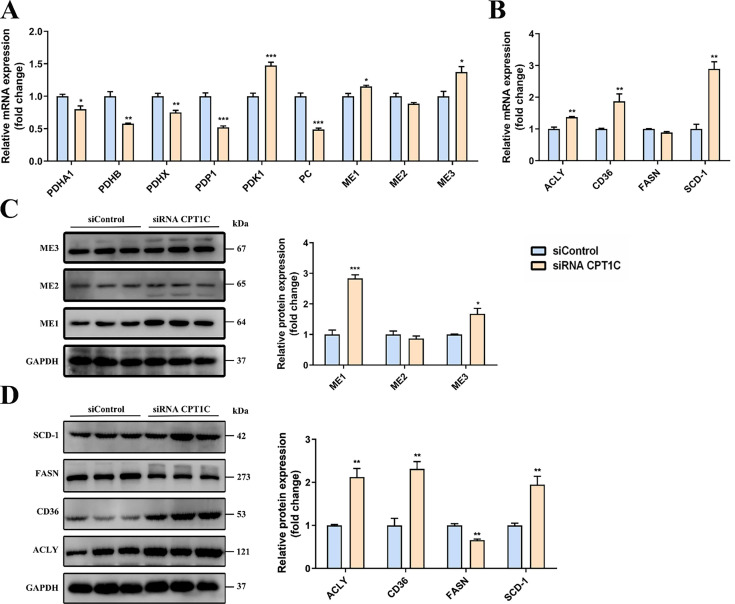
** CPT1C knockdown changes expression of genes and proteins related to citrate and fatty acid metabolism in MDA-MB-231 cells. A** Relative mRNA expression of genes related to PDH, PC and ME in MDA-MB-231 cells transfected with siRNA *CPT1C*, n=3-4. **B** Relative mRNA levels of enzymes involved in fatty acid metabolism of MDA-MB-231 cells transfected with siRNA *CPT1C*, n=3-4. **C** Western blot analysis of MEs protein levels of MDA-MB-231 cells after transfection with siRNA *CPT1C*. Left and right: images and densitometric analysis of western blot, n=3. **D** Protein levels of enzymes involved in fatty acid metabolism of MDA-MB-231 cells after transfection with siRNA *CPT1C*. Left and right: images and densitometric analysis of western blot, n=3. Data are represented as mean ± S.E.M, ^*^p < 0.05, ^**^p < 0.01, ^***^p < 0.001 versus the siControl group.

To verify the metabolic flux results, the mRNA and protein expression levels of related metabolic enzymes were determined in MDA-MB-231 cells transfected with siRNA *CPT1C*. As shown in Fig. 3A, the mRNA levels of pyruvate dehydrogenase alpha 1 (*PDHA1*), pyruvate dehydrogenase beta (*PDHB*), pyruvate dehydrogenase complex component X (*PDHX*) and pyruvate dehyrogenase phosphatase catalytic 1 (*PDP1*) were decreased after *CPT1C* knockdown, and the mRNA level of pyruvate dehydrogenase kinase 1 (*PDK1*) was increased, indicating that silencing *CPT1C* inhibited the PDH pathway activity of MDA-MB-231 cells. Moreover, the mRNA level of pyruvate carboxylase (*PC*) was decreased after *CPT1C* knockdown (Fig. 3A), suggesting that silencing *CPT1C* attenuated the transformation of pyruvate to oxaloacetate. As for the malic enzyme (ME) family, the mRNA and protein levels of ME1 and ME3 were increased upon *CPT1C* knockdown (Fig. 3A and 3C), indicating that silencing *CPT1C* enhanced malate generation from pyruvate in MDA-MB-231 cells.

For enzymes associated with fatty acid synthesis, the mRNA and protein levels of ATP citrate lyase (ACLY) and CD36 were both markedly increased after transfection with siRNA *CPT1C* in MDA-MB-231 cells (Fig. 3B and 3D), suggesting that *CPT1C* knockdown raised the endogenous and exogenous fatty acid synthesis. However, the mRNA and protein levels of fatty acid synthase (FASN) were decreased while stearoyl-Coenzyme A desaturase 1 (SCD-1) were markedly increased after silencing *CPT1C* (Fig. 3B and 3D), which was consistent with metabolic flux analysis results (Fig. 1F). In brief, *CPT1C* knockdown could affect the expression of genes and proteins related to citrate and fatty acid metabolism in MDA-MB-231 cells.

### Stearate triggers tumor cell senescence

**Figure 4:**
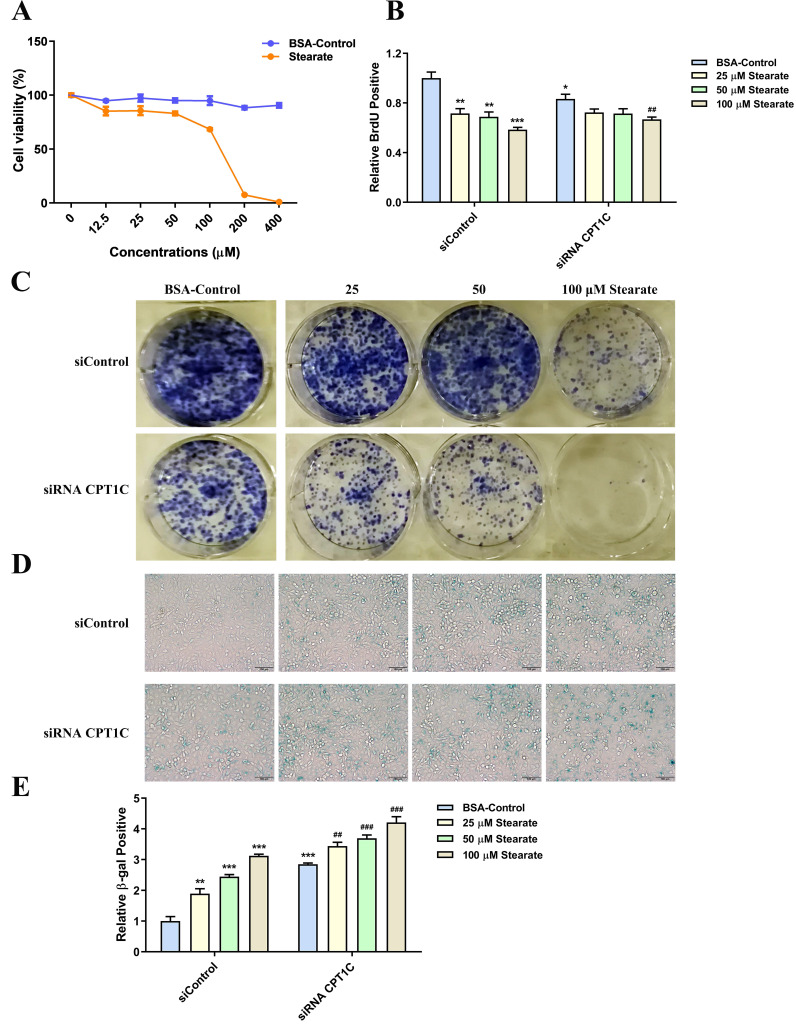
** Stearate triggers cellular senescence in MDA-MB-231 cells. A** Cellular viability profile plots of MDA-MB-231 cells cultured with a series of concentrations of BSA conjugated-stearate or BSA control, n=3. **B** BSA conjugated-stearate decreased the number of MDA-MB-231 cells transfected with siRNA *CPT1C* or siControl in replicating DNA synthesis, n=4. **C** BSA conjugated-stearate inhibited colony formation of MDA-MB-231 cells transfected with siRNA *CPT1C* or siControl. **D** Representative images of SA-β-gal activity in MDA-MB-231 cells cultured with BSA conjugated-stearate or BSA control. **E** BSA conjugated-stearate increased SA-β-gal activity of MDA-MB-231 cells transfected with siRNA *CPT1C* or siControl, n=5. Data are represented as mean ± S.E.M, ^*^p < 0.05, ^**^p < 0.01, ^***^p < 0.001 versus the siControl-BSA group,^ ##^*p* < 0.01, ^###^*p* < 0.001 versus the siRNA *CPT1C*-BSA group.

Since the synthesis rate of stearate and oleate were opposite after transfection of siRNA* CPT1C*, we further studied the effects of stearate and oleate on tumor cell proliferation and senescence. Cells were cultured in medium with dialyzed serum and different concentrations of BSA conjugated-stearate or BSA control solution. As shown in Fig. 4A, BSA conjugated-stearate at 25, 50 and 100 μM, was selected for the following experiments. Interestingly, 25, 50 and 100 μM BSA conjugated-stearate decreased the number of MDA-MB-231 cells actively replicating DNA, even in the siRNA *CPT1C* group (Fig. 4B). In addition, BSA conjugated-stearate inhibited the colony formation of MDA-MB-231 cells in a concentration-dependent manner both in the siControl and siRNA *CPT1C* groups (Fig. 4C). Besides, BSA conjugated-stearate induced senescence in the siControl group and further strengthened the senescent phenotype in siRNA *CPT1C* treated cells (Fig. 4D and 4E). Notably, BSA conjugated-stearate also inhibited proliferation and led to senescence in another tumor cell line, PANC-1 cells (Supporting Information Fig. S2A-G). Taken together, stearate could trigger cellular senescence in MDA-MB-231 and PANC-1 cells.

### Oleate causes an increase in proliferation and reverses senescent phenotype induced by silencing CPT1C in tumor cells

**Figure 5:**
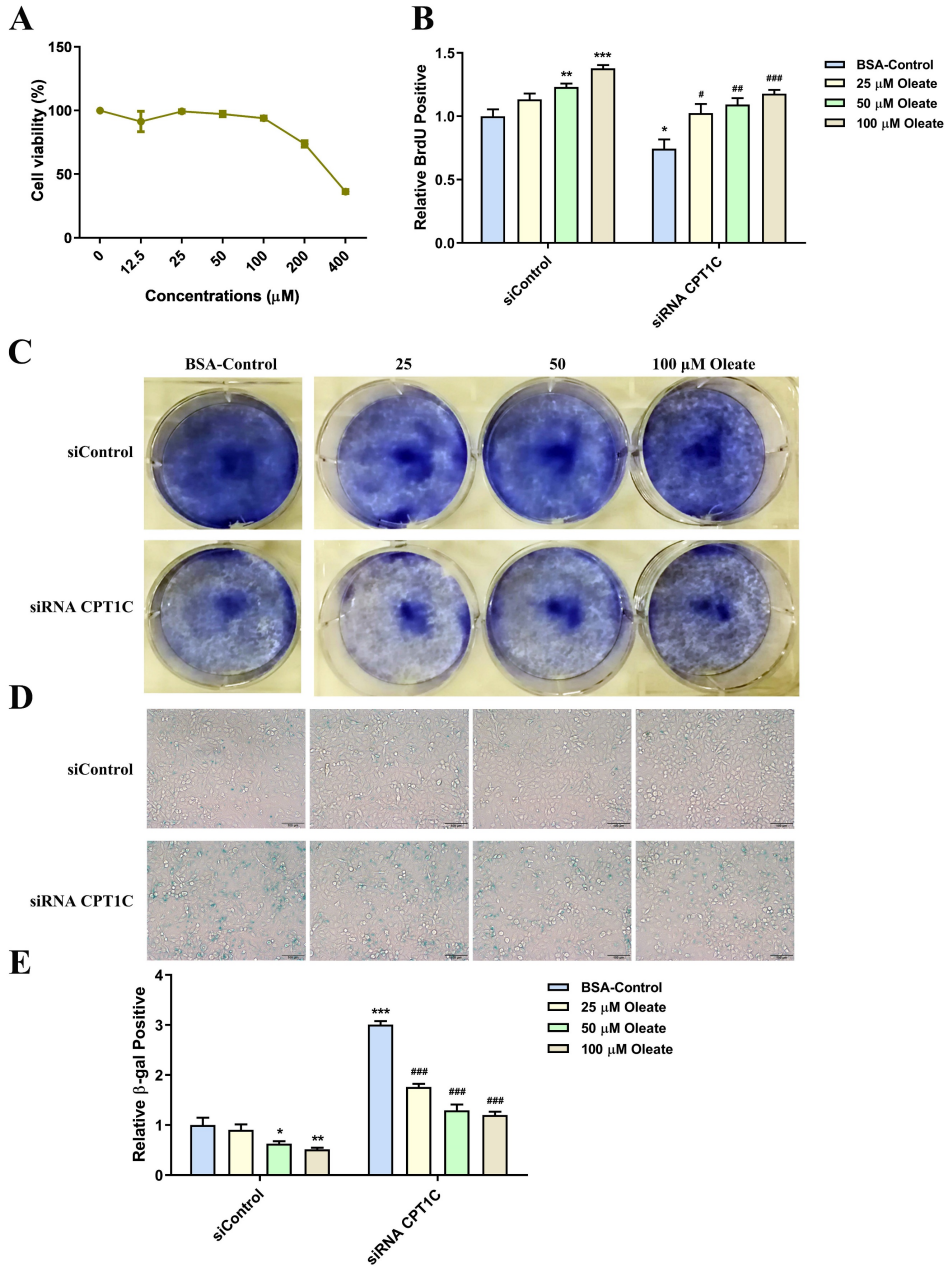
** Oleate causes an increase in proliferation and reverses senescent phenotype induced by silencing CPT1C in MDA-MB-231 cells. A** Cellular viability profile plots of MDA-MB-231 cells cultured with a series of concentrations of BSA conjugated-oleate, n=3. **B** BSA conjugated-oleate increased the number of MDA-MB-231 cells transfected with siRNA *CPT1C* or siControl in replicating DNA synthesis, n=3-5. **C** BSA conjugated-oleate strengthened colony formation of MDA-MB-231 cells transfected with siRNA *CPT1C*. **D** Representative images of SA-β-gal activity in MDA-MB-231 cells cultured with BSA conjugated-oleate or BSA control. **E** BSA conjugated-oleate decreased SA-β-gal activity of MDA-MB-231 cells transfected with siRNA *CPT1C*, n=5-6. Data are represented as mean ± S.E.M, ^*^p < 0.05, ^**^p < 0.01, ^***^p < 0.001 versus the siControl-BSA group, ^#^*p* < 0.05, ^##^*p* < 0.01, ^###^*p* < 0.001 versus the siRNA *CPT1C*-BSA group.

BSA conjugated-oleate was also used to evaluate the effect of oleate on tumor cell proliferation and senescence. And BSA conjugated-oleate at 25, 50 and 100 μM, was selected for the experiments (Fig. 5A). BSA conjugated-oleate increased the number of MDA-MB-231 cells actively replicating DNA in a concentration-dependent manner, especially in the siRNA *CPT1C* group (Fig. 5B). Besides, BSA conjugated-oleate increased the colony formation of MDA-MB-231 cells in the siRNA *CPT1C* group (Fig. 5C). Furthermore, BSA conjugated-oleate did not induce senescence in the siControl group but reduce the ratio of SA-β-gal positive cells induced by silencing *CPT1C* (Fig. 5D and 5E). Similar effects of BSA conjugated-oleate on cellular proliferation and senescence were also found in PANC-1 cells (Supporting Information Fig. S3A-E). Overall, oleate causes an increase in proliferation and reverses the senescent phenotype induced by silencing *CPT1C* in MDA-MB-231 and PANC-1 cells.

### SCD-1 inhibitor A939572 triggers cellular senescence in tumor cells

**Figure 6:**
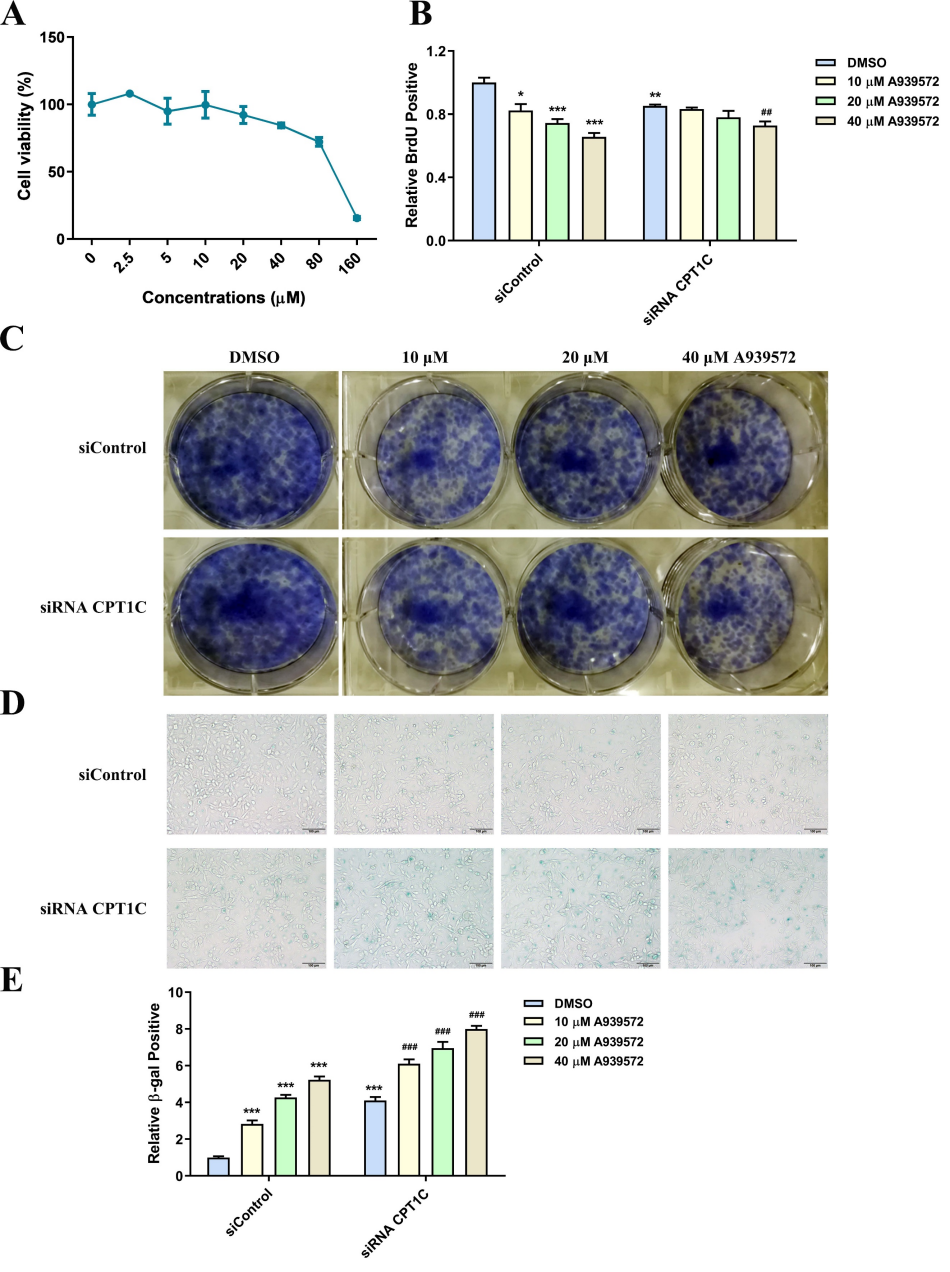
** SCD-1 inhibitor A939572 triggers cellular senescence in MDA-MB-231 cells. A** Cellular viability profile plot in MDA-MB-231 cells cultured with a series of concentrations of SCD-1 inhibitor A939572, n=3. **B** A939572 decreased the number of MDA-MB-231 cells transfected with siRNA *CPT1C* or siControl in replicating DNA synthesis, n=3-5. **C** A939572 inhibited colony formation of MDA-MB-231 cells transfected with siRNA *CPT1C* or siControl. **D** Representative images of SA-β-gal activity in MDA-MB-231 cells cultured with A939572 or DMSO. **E** A939572 increased SA-β-gal activity of MDA-MB-231 cells transfected with siRNA *CPT1C* or siControl, n=8. Data are represented as mean ± S.E.M, ^*^p < 0.05,^ **^p < 0.01, ^***^p < 0.001 versus the siControl-DMSO group, ^##^*p* < 0.01, ^###^*p* < 0.001 versus the siRNA *CPT1C*-DMSO group.

A939572 is an inhibitor of SCD-1 (the key enzyme for oleate synthesis from stearate). According to the cellular viability profile plot (Fig. 6A), A939572 at 10, 20 and 40 μM, was selected for the related experiments. Consistent with the effect of BSA conjugated-stearate, A939572 treatment significantly decreased the number of MDA-MB-231 cells actively replicating DNA in the siControl and siRNA *CPT1C* group (Fig. 6B). A939572 could also inhibit the colony formation of MDA-MB-231 cells (Fig. 6C). Furthermore, A939572 triggered senescence in the siControl group and intensified the senescent phenotype induced by silencing *CPT1C* (Fig. 6D and 6E). In addition, A939572 weakened cellular proliferation and induced senescence in PANC-1 cells (Supporting Information Fig. S4A-E). Taken together, SCD-1 inhibitor A939572 could trigger cellular senescence in MDA-MB-231 and PANC-1 cells.

## Discussion

**Figure 7:**
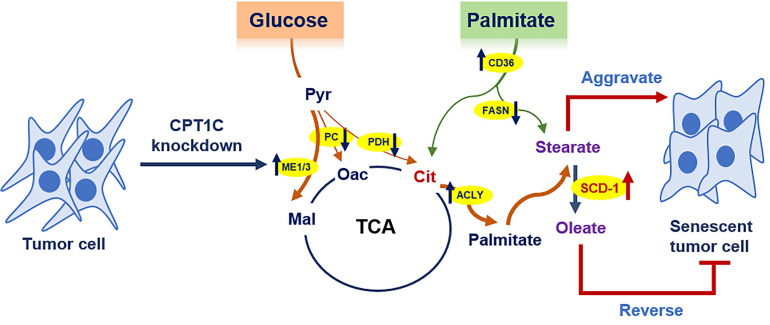
Summary of the effect of CPT1C knockdown on fatty acid metabolism during tumor cell senescence.

Cellular senescence is a barrier to malignant transformation, and senescence in tumor cells would be a more benign and favorable outcome 3. We previously reported that CPT1C is a vital regulator of tumor cell proliferation and senescence, and CPT1C downregulation can induce tumor cell senescence via mitochondria-associated dysfunction and lipotoxicity 23, 25. In this study, ^13^C-MFA results further indicated that *CPT1C* knockdown can markedly change fatty acid anabolism and catabolism in MDA-MB-231 cells. In addition, stearate and oleate were found to be involved in CPT1C-mediated tumor cell proliferation and senescence. Stearate inhibits proliferation while oleate reverses the senescent phenotype induced by silencing *CPT1C* (Fig. 7). These findings suggest that CPT1C combined with the regulation of stearate and oleate may be potential targets for cancer therapy.

CPT1C, as one subtype of the CPT family, was first reported in 2002 12. Many kinds of cancers are associated with high CPT1C expression 15, 17-21, 28, 29. CPT1C is closely related to energy supply and metabolism, and thus high-expression of CPT1C promotes cell survival and tumor development 15, 21, 30. However, the relationship between CPT1C and fatty acid metabolism during tumor cell senescence remains unknown.

In this study, ^13^C-MFA demonstrated that *CPT1C* knockdown changed anabolism and catabolism of fatty acids in MDA-MB-231 cells. The total abundance of four fatty acids were increased upon *CPT1C* knockdown, consistent with our previous report that *CPT1C* knockdown induces tumor cellular senescence via lipid accumulation 25. This was partly due to enhancing the ability of cells to uptake exogenous fatty acids induced by *CPT1C* knockdown, as evidenced by higher expression of CD36. In addition, *CPT1C* knockdown blocked the pathway by which long chain fatty acid (LCFA) are transported into mitochondria and metabolized into acetyl-CoA as revealed by the decreased abundance of labeled citrate that arose from [U-^13^C_16_] palmitate, which was consistent with previous reports that CPT1C can enhance the transport of fatty acids into mitochondria and increase FAO activity in varied tumor cells 15, 21, 31-33. However, *CPT1C* knockdown also increased the total abundance of citrate and the expression of ACLY, the first rate-limiting enzyme catalyzing the conversion of citrate to acetyl-CoA, which feeds into fatty acid synthesis in the cytoplasm 10. It was reported that extracellular citrate promotes tumor growth and metastasis 34. However, others found that citrate could inhibit tumor growth and they suggested that dietary supplementation with citrate would be beneficial as a cancer therapy 35. The dual role of citrate in cancer may depend on the concentration of citrate, since low concentrations (5 mM) citrate promotes A549 lung cancer cells growth, while 10 mM or higher concentrations of citrate inhibits cancer cell growth 35.

The increased total abundance of citrate came from the tricarboxylic acid (TCA) intermediate malate since *CPT1C* knockdown inhibited the PDH pathway and PC expression but increased the expression of ME1 and ME3. Previous studies have shown that PDH, PC and ME are all closely related to cancer development. The pyruvate dehydrogenase complex (PDC) decarboxylates pyruvate to acetyl-CoA, linking glycolysis to the TCA cycle. This process is reversible and regulated by PDK and PDP, and PDK-mediated phosphorylation is associated with many disorders of metabolic integration, including cancer 36. PC is an enzyme catalyzing the carboxylation of pyruvate to oxaloacetate, which is required for supplementing TCA cycle intermediates 37. It was reported that PC is expressed at higher level in the cancerous areas of breast tissue than non-cancerous areas, and was essential to support proliferation and invasion of MDA-MB-231 cells 38, 39. ME, including three subtypes: cytosolic NADP^+^ dependent ME1, mitochondrial NAD(P)^+^ dependent ME2 and mitochondrial NADP^+^ dependent ME3, catalyzes the reversible conversion of pyruvate to malate and is involved in NADPH production, glutamine metabolism and fatty acid synthesis. ME is highly expressed in many kinds of tumors and depletion of ME could inhibit tumor growth 40-42. In addition, the reciprocal regulation of p53 and ME modulates metabolism and senescence. P53 blocked cell metabolism and proliferation via inhibiting ME1 and ME2, and down-regulation of ME1 and ME2 could activate p53 to induce severe senescence, while overexpression of ME could inhibit cellular senescence 43.

In this study, *CPT1C* knockdown increased ME1 and ME3 expression and decreased PDH activity and PC expression, suggesting a substrate-enzyme competition among PDH, PC and ME during MDA-MB-231 cellular senescence. Increased ME expression was reported rather than PDH or PC in hypertrophied rat heart 44. On one hand, carbon units that enter oxidative metabolism from glucose via carboxylation to malate can bypass NADH generation from PDH and the first stage of the citrate cycle, reducing energy production from carbohydrate oxidation. On the other hand, ME would consume NADPH, which is necessary for triglyceride (TG) synthesis 44. We previously showed that *CPT1C* knockdown induces FA and TG accumulation, thereby triggering lipotoxicity 25. Thus, the increased ME1 and ME3 expression may be the result of feedback from cellular lipid accumulation and lipotoxicity, and the expression of NAD(P)^+^dependent ME2 was not changed significantly upon *CPT1C* knockdown.

The current results indicated that *CPT1C* knockdown may increase the uptake of exogenous fatty acids via CD36 and endogenous fatty acids synthesis by ACLY and ME. However, the expression of FASN was decreased while the SCD-1 was increased upon *CPT1C* knockdown, consistent with the decreased stearate synthesis rate and increased oleate synthesis rate. It is noteworthy that stearate is a saturated fatty acid and oleate is an unsaturated fatty acid. Saturated and unsaturated fatty acids play different roles in aging and cell fate. It has been reported that anti-aging metabolites would be depleted by palmitate but increased by oleate 45. High palmitate results in MDA-MB-231 cell death. By contrast, high-level oleate can promote cell proliferation, migration and invasion 46.

Since stearate and palmitate have similar structures, we further examined the effects of stearate and oleate on cell proliferation and senescence. The results showed that stearate triggered cellular senescence while oleate caused an increase in proliferation and reversed the senescent phenotype induced by silencing *CPT1C* in MDA-MB-231 and PANC-1 cells. It was reported that oleate stimulated cell proliferation while palmitate inhibited cell proliferation and induced apoptosis 47-49. Palmitate enhances early cardiolipin turnover and reduces the levels of mitochondrial phospholipid. Cosupplementation of oleate restores cardiolipin levels and inhibits palmitate-induced apoptosis 47. Supplementation of oleate induces TG accumulation and is well tolerated, but excess palmitate is poorly incorporated into triglyceride. Oleate can rescue palmitate-induced apoptosis via channeling palmitate into triglyceride pools and just induce lipotoxicity in the setting of impaired TG synthesis 47, 50. We previously reported that *CPT1C* knockdown could decrease cardiolipin and increase FA and TG, triggering lipotoxicity and mitochondrial functional injury 22, 25. Thus, there may be a "self-help" and "compensatory" feedback in cells, reducing saturated stearate and increasing unsaturated oleate, to resist senescence induced by silencing *CPT1C*.

Finally, we selected an inhibitor of SCD-1 (an enzyme converts stearate to oleate) to further confirm the effects of stearate and oleate on cellular proliferation and senescence. The SCD-1 inhibitor A939572 had similar effects with stearate on MDA-MB-231 and PANC-1 cells. It was reported that high SCD-1 expression is associated with shorter survival in breast cancer patients 51. In addition, elevated SCD-1 expression was also observed in human prostate cancer, hepatocellular carcinoma (HCC) and clear cell renal cell carcinoma (ccRCC). SCD-1 inhibition can decrease cell viability and inhibit tumor growth 52-54. Consistent with these findings, our results showed that elevating the stearate/oleate ratio via inhibition of SCD-1 can depress tumor cell proliferation and stimulate senescence.

In conclusion, the current ^13^C-MFA study demonstrated that *CPT1C* knockdown notably alters fatty acid anabolism and catabolism in MDA-MB-231 cells, especially the synthesis rate of stearate was decreased and oleate was increased. Furthermore, stearate and oleate are involved in CPT1C-mediated tumor cell proliferation and senescence. Stearate inhibits proliferation while oleate reverses the senescent phenotype induced by silencing *CPT1C*, suggesting that the opposite synthesis rate of stearate and oleate is a strategy to resistance cellular senescence. Thus, these results revealed the regulation of stearate/oleate rate via inhibition of SCD-1 could be an additional strategy with depletion of CPT1C to induce tumor cell senescence.

## Supplementary Material

Supplementary figures and tables.

## Abbreviations

CPT1C: carnitine palmitoyltransferase 1C
^13^C-MFA: ^13^C-metabolic flux analysis
CPT: carnitine palmitoyltransferase
PPARα: peroxisome proliferator-activated receptor α
ERRα: estrogen-related receptor α
FA: fatty acid
siRNA: small interfering RNA
DMEM: Dulbecco's modified Eagle's medium
FBS: fetal bovine serum
BSA: bovine serum albumin
FAMEs: fatty acid methyl esters
GC/MS: gas chromatography/mass spectrometry
EI: electron impact
eV: electronvolts
m/z: mass-to-charge ratio
SDS-PAGE: sodium dodecyl sulfate polyacrylamide gel electrophoresis
PDHA1: pyruvate dehydrogenase alpha 1
PDHB: pyruvate dehydrogenase beta
PDHX: pyruvate dehydrogenase complex component X
PDP1: pyruvate dehyrogenase phosphatase catalytic 1
PDK1: pyruvate dehydrogenase kinase 1
PC: pyruvate carboxylase
ME: malic enzyme
ACLY: ATP citrate lyase
FASN: fatty acid synthase
SCD-1: stearoyl-Coenzyme A desaturase 1
LCFA: long chain fatty acid
TCA: tricarboxylic acid
PDC: pyruvate dehydrogenase complex
TG: triglyceride
HCC: hepatocellular carcinoma
ccRCC: clear cell renal cell carcinoma
AcCoA: acetyl-CoA
αKG: α-ketoglutarate
Cit: citrate
Fum: fumarate
Mal: malate
Oac: oxaloacetate
Suc: succinate
Pyr: pyruvate
