# Supplementary material for: Metabolic Flux Analysis Reveals the Roles of Stearate and Oleate on CPT1C-mediated Tumor Cell Senescence
Source: Int J Biol Sci. 2023 Apr 9;19(7):2067–80. doi: 10.7150/ijbs.80822 (PMC10158022; doi:10.7150/ijbs.80822)
Supplement: Supplementary file 1 — Supplementary figures and tables. [file ijbsv19p2067s1.pdf]

Supplementary Materials

Supplementary Figures

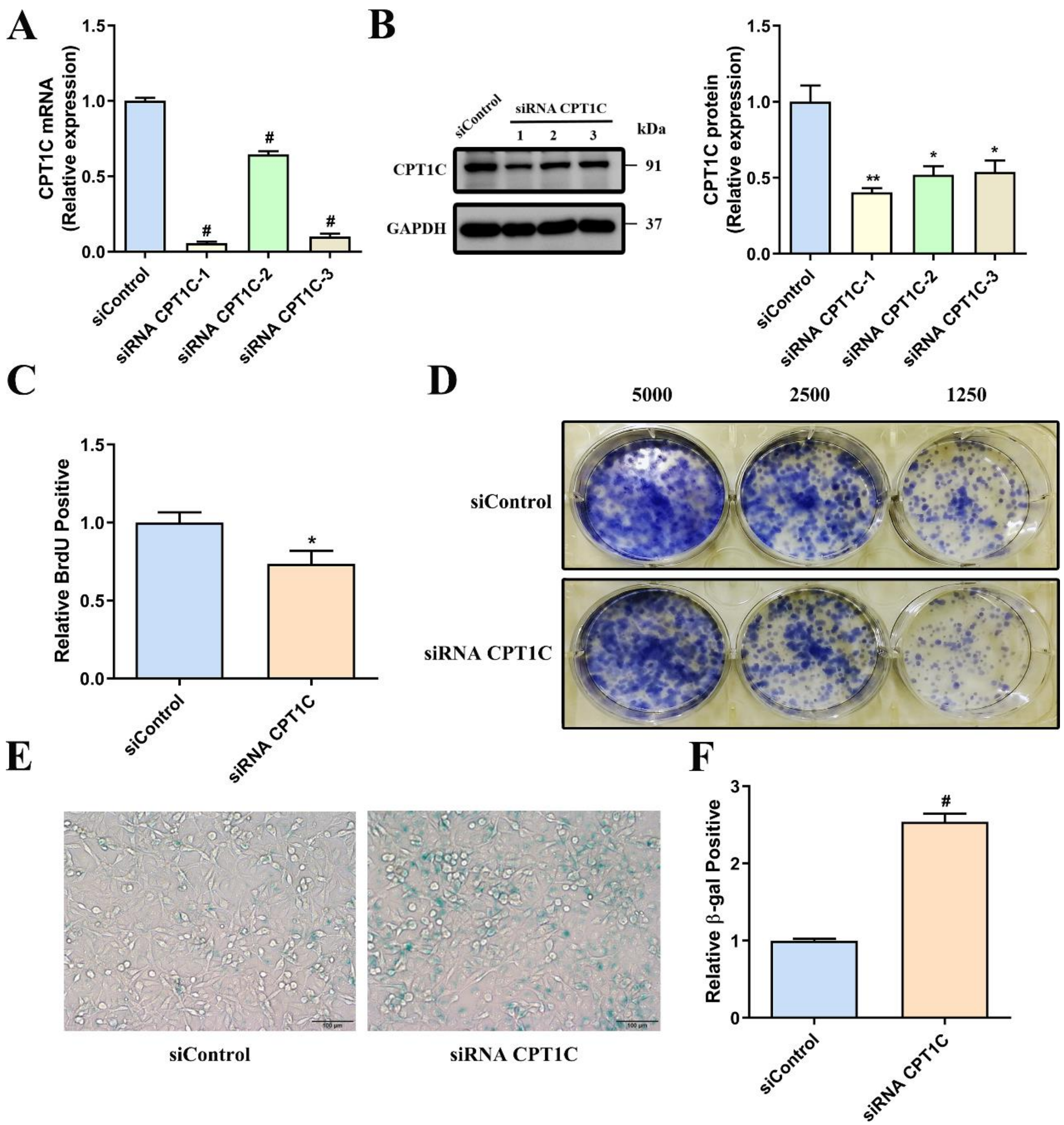

Figure S1. CPT1C knockdown triggers cellular senescence in MDA-MB-231 cells.

A Relative *CPT1C* mRNA expression was measured post-transfection with siRNA *CPT1C* in MDA-MB-231 cells, n=4. **B** CPT1C protein expression was detected in MDA-MB-231 cells after transfection

with siRNA *CPT1C*. Left panel of B shows images and right panel of B shows densitometric analysis of western blot, n=3. **C** The number of MDA-MB-231 cells transfected with siRNA *CPT1C* was decreased in replicating DNA synthesis measured by BrdU incorporation, n=5. **D** The colony formation of MDA-MB-231 cells was inhibited after transfection with siRNA *CPT1C* in a concentration-dependent manner. **E** Representative images of SA-β-gal activity of MDA-MB-231 cells transfected with siRNA *CPT1C* or siControl. **F** MDA-MB-231 cells transfected with siRNA *CPT1C* showed higher SA-β-gal activity, n=5. Data are represented as mean ± S.E.M, $^{*}p < 0.05$, $^{**}p < 0.01$, $^{\#}p < 0.0001$ versus the siControl group.

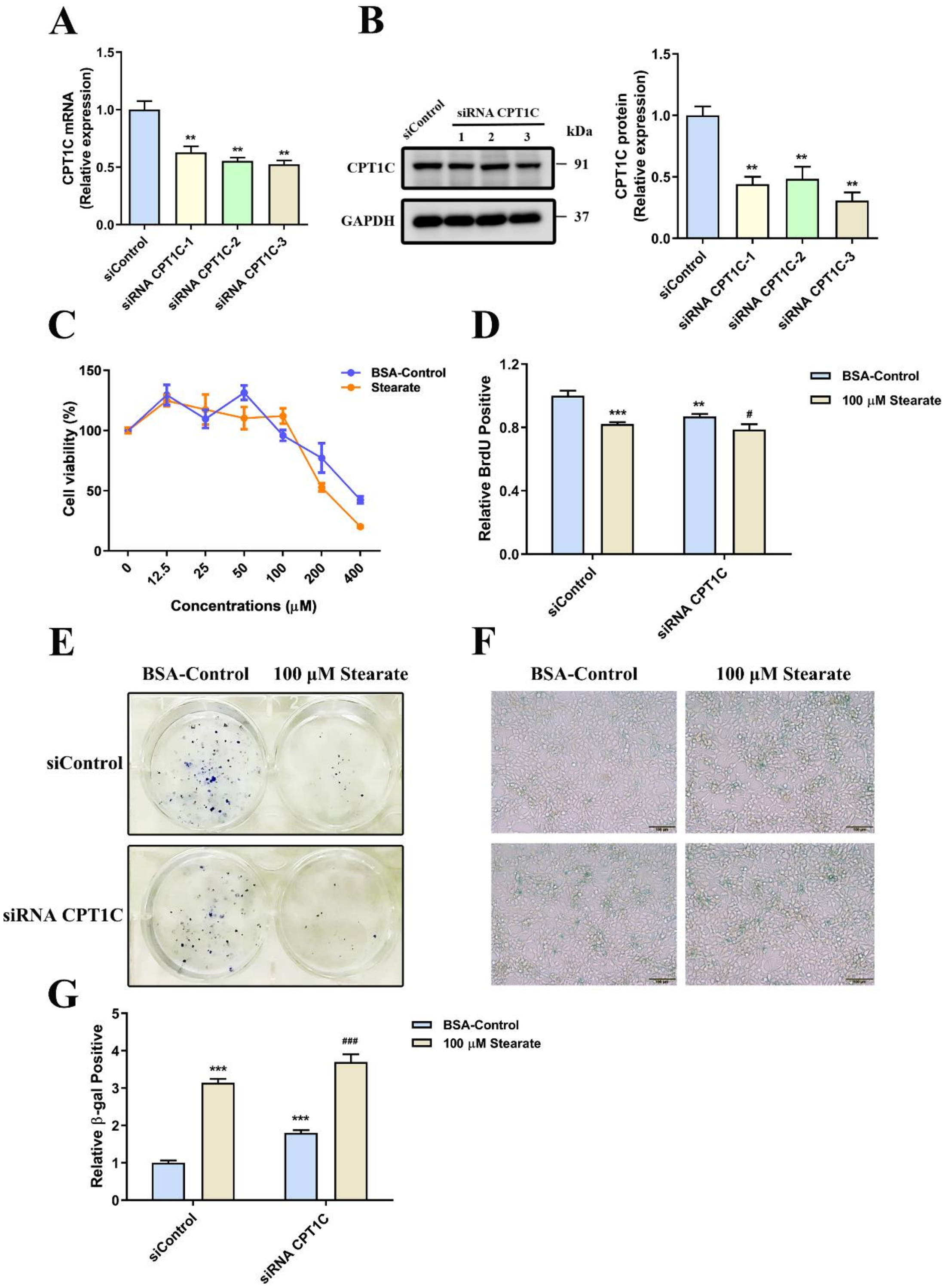

Figure S2. Stearate confers to cellular senescence in PANC-1 cells.

A Relative *CPT1C* mRNA expression in PANC-1 cells transfected with siRNA *CPT1C*, n=3-4. **B** CPT1C protein level in PANC-1 cells after transfection with siRNA *CPT1C*. (Left panel of **B**) western

blot images and (right panel of **B**) densitometric analysis, n=3-4. **C** Cellular viability profile plots in PANC-1 cells cultured with a series of concentrations of BSA conjugated-stearate or BSA control, n=3. **D** BSA conjugated-stearate induced PANC-1 cells lower proliferation, n=4-5. **E** BSA conjugated-stearate reduced the colony formation ability of PANC-1 cells transfected with siRNA *CPT1C* or siControl. **F** Representative images of SA-β-gal activity in PANC-1 cells cultured with BSA conjugated-stearate or BSA control. **G** BSA conjugated-stearate increased SA-β-gal activity of PANC-1 cells transfected with siRNA *CPT1C* or siControl, n=5. Data are represented as mean ± S.E.M, $^{**}p < 0.01$, $^{***}p < 0.001$ versus the siControl-BSA group, $^{\#}p < 0.05$, $^{\#\#\#}p < 0.001$ versus the siRNA *CPT1C*-BSA group.

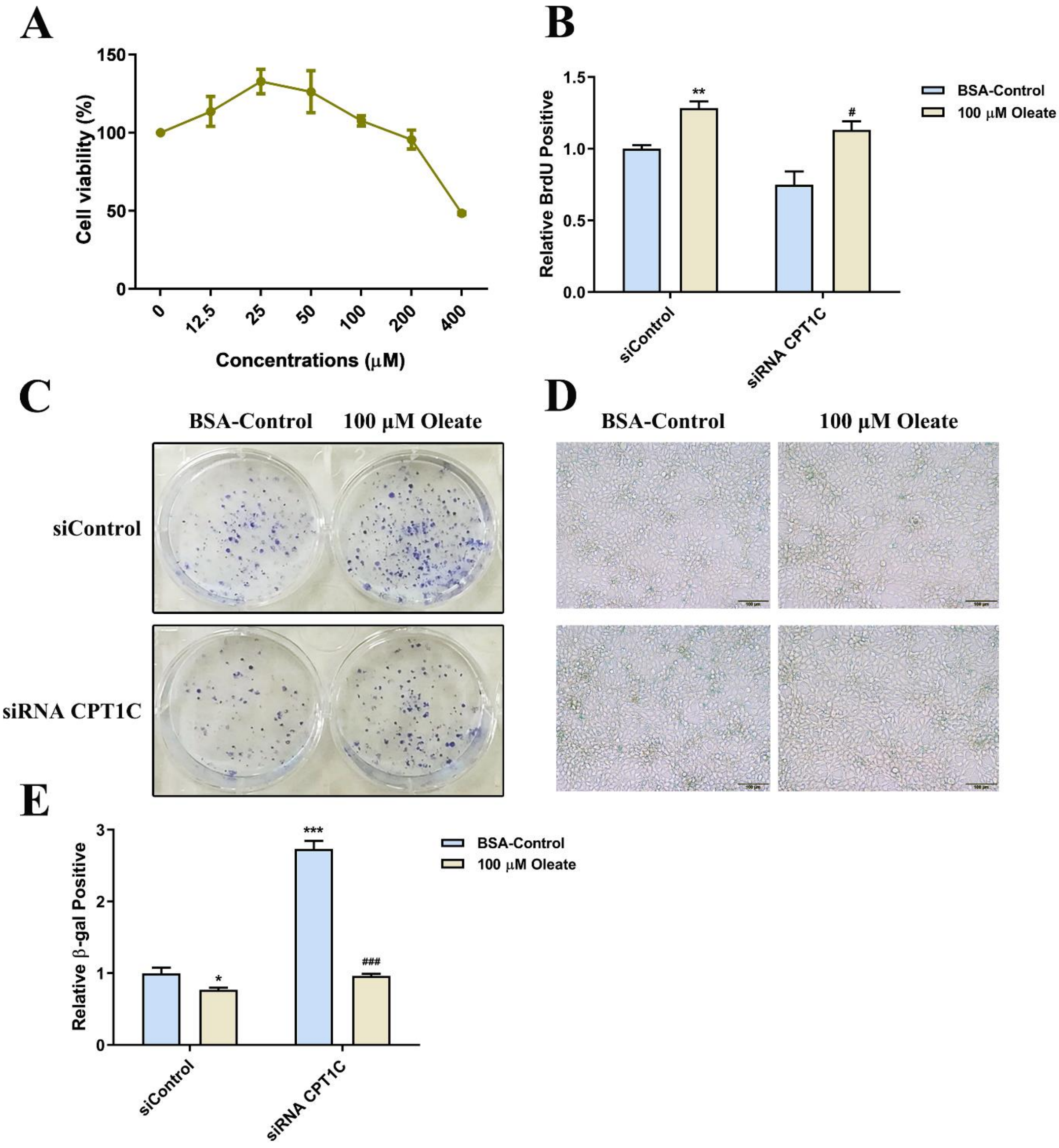

Figure S3. Oleate causes an increase in proliferation and reverses senescent phenotype induced by silencing CPT1C in PANC-1 cells.

A Cellular viability profile plots in PANC-1 cells cultured with a series of concentrations of BSA conjugated-oleate, n=3. **B** BSA conjugated-oleate induced PANC-1 cells higher proliferation, n=3-4. **C** BSA conjugated-oleate up-regulated the colony formation ability of PANC-1 cells transfected with

siRNA *CPT1C* or siControl. **D** Representative images of SA-β-gal activity in PANC-1 cells cultured with BSA conjugated-oleate or BSA control. **E** BSA conjugated-oleate decreased SA-β-gal activity of PANC-1 cells transfected with siRNA *CPT1C*, n=5. Data are represented as mean ± S.E.M, $^{*}p < 0.05$, $^{**}p < 0.01$, $^{***}p < 0.001$ versus the siControl-BSA group, $^{\#}p < 0.05$, $^{\#\#\#}p < 0.001$ versus the siRNA *CPT1C*-BSA group.

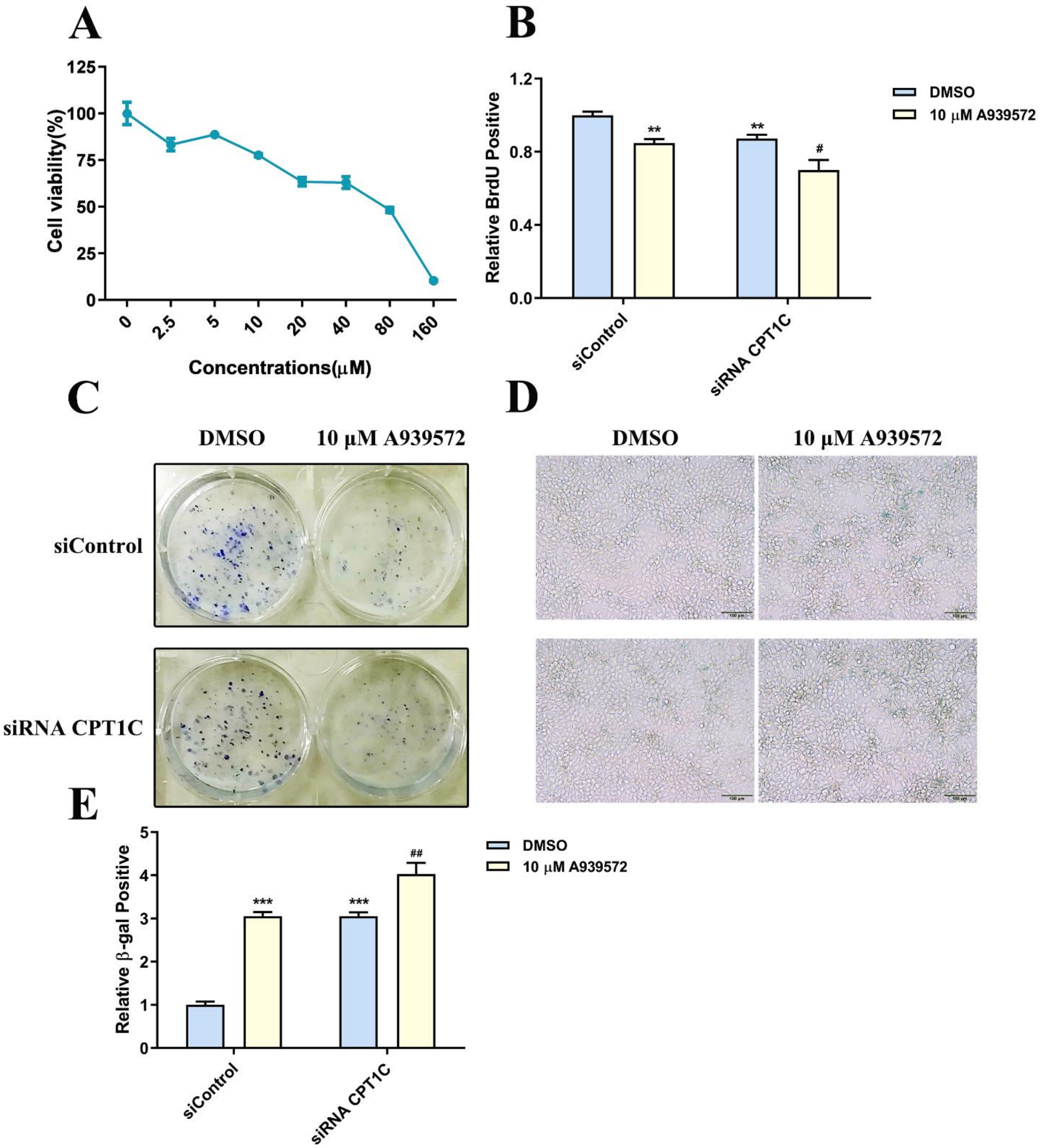

Figure S4. SCD-1 inhibitor A939572 confers to cellular senescence in PANC-1 cells.

A Cellular viability profile plot in PANC-1 cells cultured with a series of concentrations of SCD-1 inhibitor A939572, n=3. **B** A939572 induced PANC-1 cells lower proliferation, n=3-4. **C** A939572 down-regulated the colony formation ability of PANC-1 cells transfected with siRNA *CPT1C* or

siControl. **D** Representative images of SA-β-gal activity in PANC-1 cells cultured with A939572. **E** A939572 increased SA-β-gal activity of PANC-1 cells transfected with siRNA *CPT1C* or siControl, n=6. Data are represented as mean ± S.E.M, $^{**}p < 0.01$, $^{***}p < 0.001$ versus the siControl-DMSO group, $^{\#}p < 0.05$, $^{\#\#}p < 0.01$ versus the siRNA *CPT1C*-DMSO group.

Supplementary Tables

Table S1. Sequences of RNAi assays.

RNAi Name	Species Specificity	Sequences
siRNA *CPT1C*-1	Human	5`-GCCAUGAUCGCUGGUUUGA dTdT-3`
siRNA *CPT1C*-2	Human	5`-CGGCCAUGACUCGCUUAUU dTdT-3`
siRNA *CPT1C*-3	Human	5`-ACCUGUUUGCGCUGUACAU dTdT-3

Table S2. Sequence of primers for quantitative real-time PCR analysis.

Name	NCBI Gene ID	Species Specificity	Sequences of Primers
ACTB	60	Human	forward 5′- CCTTGCACATGCCGGAG-3′
			reverse 5′-GCACAGAGCCTCGCCTT-3′

CPT1C	126129	Human	forward 5′-GGATGGCACTGAAGAGGAAA-3′
			reverse 5′-TCCTGGAAAAGGCATCTCTC-3′
PDHA1	5160	Human	forward 5′-TGGTAGCATCCCGTAATTTTGC-3′
			reverse 5′-ATTCGGCGTACAGTCTGCATC-3′
PDHB	5162	Human	forward 5′-AAGAGGCGCTTTCACTGGAC-3′
			reverse 5′-ACTAACCTTGTATGCCCCATCA-3′
PDHX	8050	Human	forward 5′-TTGGGAGGTTCCGACCTGT-3′
			reverse 5′-CAACCACTCGACTGTCACTTG-3′
PC	5091	Human	forward 5′-ACAGAGGTGAGATTGCCATCC-3′
			reverse 5′-CACTGCATCTACGTTGTTCTCC-3′
PDP1	54704	Human	forward 5′-TGTGAACTGAGCAGGATCTATGG-3′
			reverse 5′-GGAATGTACGATGAGGAACAACA-3′
PDK1	5163	Human	forward 5′-CTGTGATACGGATCAGAAACCG-3′
			reverse 5′-TCCACCAAACAATAAAGAGTGCT-3′
ME1	4199	Human	forward 5′-CTGCTGACACGGAACCCTC-3′

			reverse 5′-GATCTCCTGACTGTTGAAGGAAG-3′
ME2	4200	Human	forward 5′-ATGTTGTCCCGGTTAAGAGTAGT-3′
			reverse 5′-ACCAAGCATTTGTCGTTCTTGT-3′
ME3	10873	Human	forward 5′-TGAAGAAGCGCGGATACGATG-3′
			reverse 5′-GAAAGCAGGGCGGGATTAGG-3′
ACLY	47	Human	forward 5′-ATCGGTTCAAGTATGCTCGGG-3′
			reverse 5′-GACCAAGTTTTCCACGACGTT-3′
CD36	948	Human	forward 5′-AAGCCAGGTATTGCAGTTCTTT-3′
			reverse 5′-GCATTTGCTGATGTCTAGCACA-3′
FASN	2194	Human	forward 5′-AAGGACCTGTCTAGGTTTGATGC-3′
			reverse 5′-TGGCTTCATAGGTGACTTCCA-3′
SCD-1	6319	Human	forward 5′-TTCCTACCTGCAAGTTCTACACC-3′
			reverse 5′-CCGAGCTTTGTAAGAGCGGT-3′

Table S3. Related to Figures 2-3. Metabolite fragments used for GC/MS analysis.

Metabolite	Carbons	Derivatization	m/z	Fragments for integration
α-Ketoglutarate	1,2,3,4,5	tBDMS	346	$C_{14}H_{28}O_5NSi_2$
Lactate	1,2,3	tBDMS	261	$C_{11}H_{25}O_3Si_2$
	2,3		233	$C_{10}H_{25}O_2Si_2$
Citrate	1,2,3,4,5,6	tBDMS	459	$C_{20}H_{39}O_6Si_3$
Fumarate	1,2,3,4	tBDMS	287	$C_{12}H_{23}O_4Si_2$
Malate	1,2,3,4	tBDMS	419	$C_{18}H_{39}O_5Si_3$
Norvaline	1,2,3,4,5	tBDMS	288	$C_{13}H_{30}O_2NSi_2$
Pyruvate	1,2,3	tBDMS	174	$C_6H_{12}O_3NSi$
Succinate	1,2,3,4	tBDMS	289	$C_{12}H_{25}O_4Si_2$
Oleate	1-18	FAME	296	$C_{19}H_{36}O_2$
Palmitate	1-16	FAME	270	$C_{17}H_{34}O_2$
Stearate	1-18	FAME	298	$C_{19}H_{38}O_2$
